# Fast 3D YOLOv3 based standard plane regression of vertebral bodies in intra-operative CBCT volumes

**DOI:** 10.1117/1.JMI.10.3.034503

**Published:** 2023-05-19

**Authors:** Sebastian Doerrich, Florian Kordon, Felix Denzinger, Jan S. El Barbari, Maxim Privalov, Sven Y. Vetter, Andreas Maier, Holger Kunze

**Affiliations:** a Otto-Friedrich University Bamberg, Chair of Explainable Machine Learning, Bamberg, Germany; b Friedrich-Alexander University Erlangen-Nuremberg, Pattern Recognition Lab, Erlangen, Germany; c Siemens Healthcare GmbH, Forchheim, Germany; d Friedrich-Alexander University Erlangen-Nuremberg, Erlangen Graduate School in Advanced Optical Technologies, Erlangen, Germany; e BG Trauma Center Ludwigshafen, Ludwigshafen, Germany

**Keywords:** multiplanar reconstruction, orthopedics, flat panel computed tomography, plane regression

## Abstract

**Purpose:**

Mobile C-arm systems represent the standard imaging devices within the field of spine surgery. In addition to 2D imaging, they allow for 3D scans while preserving unrestricted patient access. For viewing, the acquired volumes are adjusted such that their anatomical standard planes align with the axes of the viewing modality. This difficult and time-consuming step is currently performed manually by the leading surgeon. This process is automatized within this work to improve the usability of C-arm systems. Thereby, the spinal region consisting of multiple vertebrae and the standard planes of all vertebrae being of interest to the surgeon need to be taken into account.

**Approach:**

An object detection algorithm based on the you only look once version 3 architecture, adapted to 3D inputs, is compared with a segmentation-based approach employing a 3D U-Net. Both algorithms are trained on a dataset of 440 and tested on 218 spinal volumes.

**Results:**

Although the detection-based algorithm is slightly inferior concerning the detection (91% versus 97% accuracy), localization (1.26 mm versus 0.74 mm error) and alignment accuracy (5.00 deg versus 4.73 deg error), it outperforms the segmentation-based one in terms of speed (5 s versus 38 s).

**Conclusions:**

Both algorithms show similar good results. However, the speed gain of the detection-based algorithm, resulting in a run time of 5 s, makes it more suitable for usage in an intra-operative scenario.

## 1 Introduction

Intra-operative 3D imaging has become a valuable tool in recent years, allowing the 3D assessment of surgery in the operating room. For that, mobile C-arm systems acquire several hundred x-ray images with a flat panel detector on a circular trajectory around the patient intra-operatively and are used to compute cone-beam computed tomography (CBCT) volumes. By viewing these volumes, a surgeon can assess implant position and fracture reduction. This allows for the treatment of implant malpositions or remaining fractures already during the intervention and thus reduces the need for subsequent revision surgeries.^1^

**Fig. 1:**
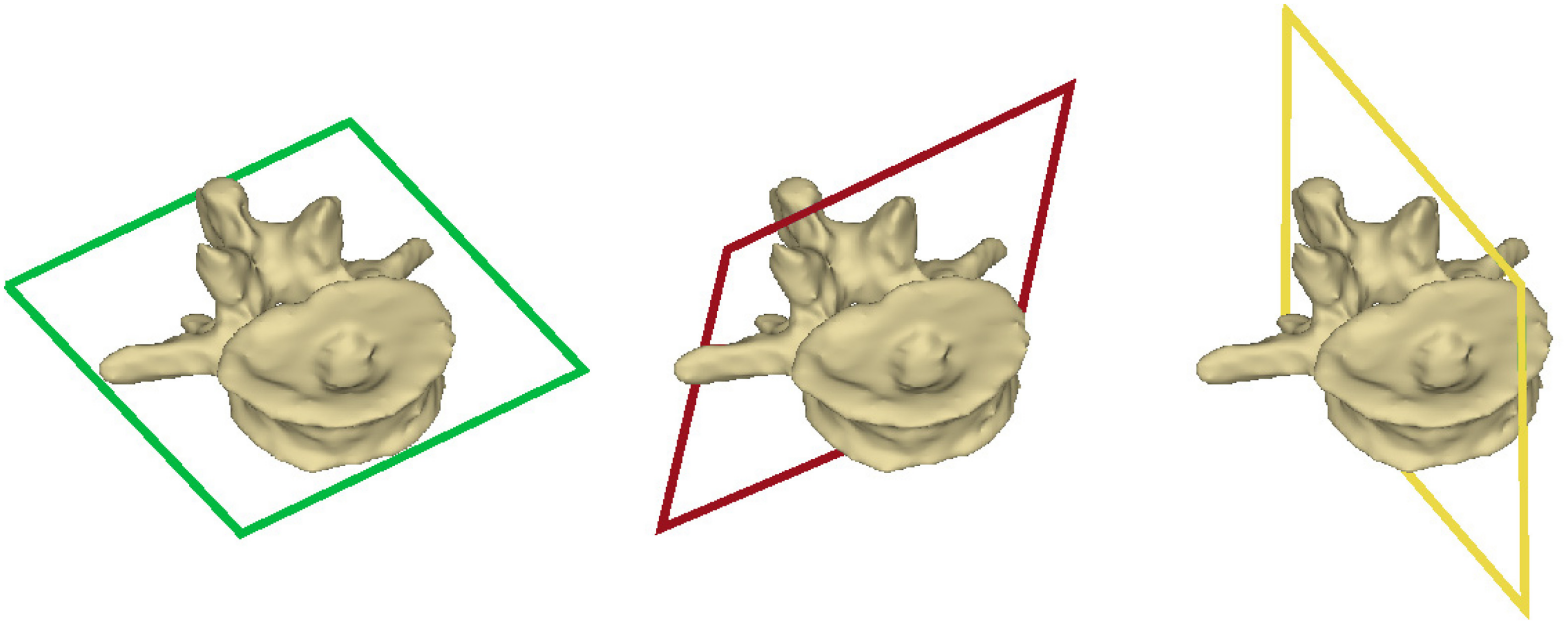
Axial (green), coronal (red), and sagittal (yellow) standard planes of a single vertebra.

An essential task in assessing 3D images is the generation of the so-called standard planes (see Fig. 1). The standard planes are used by physicians to obtain a standardized 3D volume view of the anatomical structure, showing its key features.^2^ They help to facilitate the evaluation process as well as reduce the risk of overlooking damages. For the spine, the standard planes are orthogonal to each other. The individual planes are called the axial, coronal, and sagittal planes.^3^

**Fig. 2:**
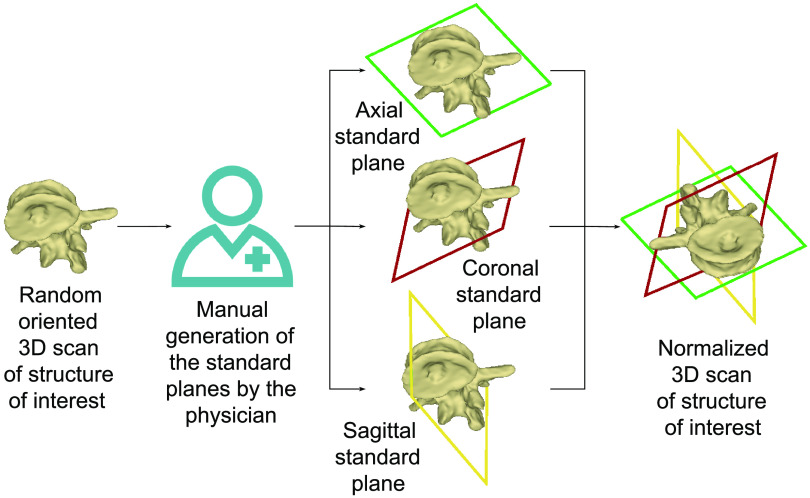
Current manual standard planes adjustment that is needed to obtain a standardized view of the structure of interest from the 3D CBCT scan.

In contrast to computed tomography (CT) produced images, the images created by mobile C-arm systems lack information about the relative position of the patient and the device. This deficiency makes a retrospective adjustment of the standard planes necessary. Until now, the surgeon has needed to perform this adjustment manually. Although this action can be interpreted as a normalization procedure, it depends on the physician and leaves leeway for mistakes.^4^ The process includes finding the center of the vertebra and adjusting the orientations of the standard planes. This step can take up to 210 s depending on the anatomical region and the experience level of the surgeon.^5^^,^^6^ Hence, it increases the overall surgery duration. An illustration of this process can be seen in Fig. 2. Spine surgeries typically involve the assessment and handling of multiple vertebrae. Moreover, a CBCT volume can include up to 10 vertebrae. Thus, the plane adjustment needs to be performed several times. Therefore, fast automation of the vertebral body detection and standard plane regression for CBCT volumes is needed to accelerate and standardize this process and reduce the reader dependency.

Recent literature has proposed different strategies for automatic standard plane adjustment. Lu et al.^7^ published an algorithm using the random forest classifier to regress standard planes in 3D echocardiography volumes automatically. They achieved a mean standard plane error of 3.7 mm and 11.3 deg, respectively. H. Chen et al.^8^ used a knowledge transferred recurrent neural network to detect standard planes from ultrasound (US) videos independent of the underlying anatomical region.

Lu et al.^9^ developed a convolutional neural network (CNN) in the form of an iterative transformation network for identifying the standard planes within 3D fetal brain US images. The proposed algorithm achieved an error of 3.83 mm and 12.7 deg. Dou et al.^10^ proposed a reinforcement learning framework to localize the standard planes in 3D fetal brain US images. In Ref. 6, our group introduced a PoseNet-like architecture estimating one triplet of standard planes in two different body regions within 3D C-arm images. We showed that the direct regression of position and spanning vectors of the planes is superior to Euler angle and quaternion representation. The inference can be done within a second.

All of these algorithms were developed for problems for which a single combination of standard planes is of interest. However, for the spinal region, a localization task for the different vertebrae needs to be incorporated.

Shi et al.^11^ proposed a two-step algorithm to localize and segment vertebral bodies in CT images using a combination of a 2D and a 3D U-net.^12^^,^^13^ The proposed algorithm achieved segmentation results with an identification rate of about 92% in under 20 s. However, it could only segment intact vertebral bodies in the absence of metal implants.

In contrast, Thomas et al.^2^ developed an assistance system that provides a side-to-side view of a patient’s ankle joints for visual comparison and evaluation for intra-operative CBCT volumes. In a first step, the ankle’s position was localized by segmenting a sphere at the upper ankle joint in a coarse volume. Then, in an region-of-interest around a single upper ankle joint, flat cylinders with the orientation of the standard planes were segmented. Based on these cylinders, the parameters for the standard planes were calculated. The approach achieved a median position-to-plane error of 0.73 mm and a mean angular error for the plane normals between 2.98 deg and 3.71 deg in under 15 s of execution time. However, the approach has shown to be susceptible to offsets of the ankle from the isocenter. For the spine, the execution time will increase because more instances are present, and thus the costly step for the plane parameter regression needs to be done repeatedly.

Alternatively, the standard plane regression can be achieved based on a preceded bounding box prediction. Jaeger et al.^14^ developed an algorithm for object detection and bounding box regression using semantic segmentation in 2D as well as 3D.

Another possibility for object detection in bounding box prediction is the “you only look once” (YOLO) algorithm. Initially introduced in Redmon et al.^15^ in 2015 and in the meantime developed further, YOLO is one of the most frequently used real-time object detection models.^16^ The goal was to develop a detection system that can compete with the human visual system in speed and accuracy.^15^ Apart from the prevalent object detectors that re-purpose classifiers to perform detection, YOLO reframes object detection as a single regression problem. There is no longer any need for slow sliding window approaches such as in deformable parts models or slow region proposal algorithms such as in region-based CNNs. The detection is executed on full images in one single evaluation and therefore achieves real-time performance.

In this work, we describe a first-of-its-kind approach to automatically localize vertebral bodies and regress their associated standard planes within intra-operative 3D CBCT volumes. For this purpose, we present a novel algorithm based on the YOLOv3 architecture and adapted for 3D inputs that regresses the standard planes in addition to the vertebrae surrounding bounding box parameters. To evaluate the model, we create a reference model by adapting the approach of Thomas et al.^2^ to enable the regression of the standard planes for multiple vertebrae within an input volume. We further demonstrate the usability of both algorithms for the automatic standard plane regression of vertebral bodies in intra-operative CBCT volumes through proof-of-concept experiments. The experiments show that the proposed YOLO-based algorithm yields comparable results to the reference approach while accelerating the plane regression by a factor of 8. This makes it more suitable for usage in an intra-operative scenario than the adapted approach of Thomas et al.^2^ The main contributions of this paper can be summarized as follows.

• modification of the segmentation-based approach of Thomas et al.^2^ for the regression of the anatomical standard planes in the human ankle (single point of interest) to work for an unknown amount of vertebrae (multiple points of interest) instead.
• Development of a novel Yolov3-based approach to automatically localize and regress the anatomical standard planes of vertebral bodies within intra-operative 3D CBCT volumes.
• Demonstration of the proposed method’s benefit in comparison with the segmentation-based approach on a unique dataset containing standard plane annotated CBCT volumes.

The remainder of this paper is structured as follows. Section 2 contains the description of both algorithms followed by an explanation of the experimental setup. The results are presented in Sec. 4. A discussion of the results concludes the paper.

## 2 Material and Methods

**Fig. 3:**
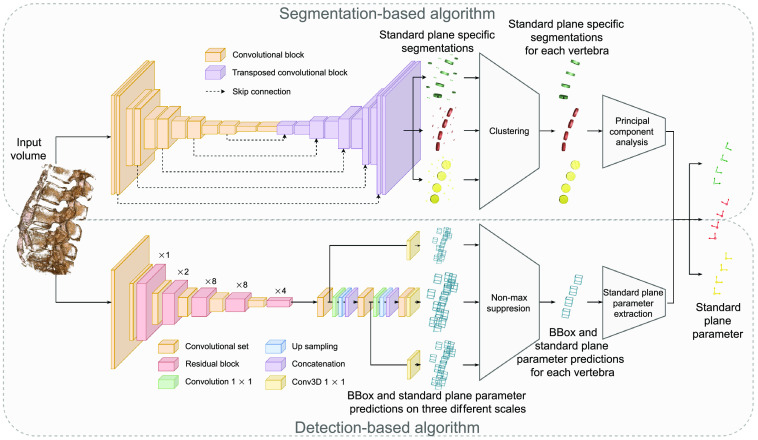
Segmentation- and detection-based approaches.

To address the task at hand, two different approaches were developed. The first approach (detection-based approach) aims to extract the standard planes for each vertebra by utilizing a preceding detection of their vertebral bodies. the second approach (segmentation-based approach) is designed to identify the standard planes for each vertebra by utilizing a preceding simplified segmentation of their vertebral bodies. The underlying idea of both approaches is visualized in Fig. 3, and their procedures are described below in more detail.

### 2.1 Detection-Based Algorithm (YOLO)

Originally, YOLOv3 was designed for detecting objects within 2D images of size 416×416  pixels by predicting the axes aligned bounding boxes. The network uses convolutional blocks (CNN) together with residual blocks (RES) for feature learning, detection blocks (SPD) for object detection, and upsampling blocks (UPS) to allow object detection at three different scales. Applying YOLOv3 on the input size mentioned above results in the first scale having a size of 13×13  pixels, the second scale of 26×26  pixels, and the third scale of 52×52  pixels. For current computer systems, these dimensions are not feasible for the 3D variant. Neither running the network’s training nor the inference on patches of size 416×416×416  voxels would be possible without exploiting disproportionate powerful processing units. The resulting high hardware costs would prevent the algorithm’s usage within the intra-operative environment. Hence, to allow the processing of 3D input volumes, the original YOLOv3 architecture was first scaled from 2D to 3D by adding an additional depth dimension for each internal operation (e.g., 2D convolution → 3D convolution). Further, to maintain the resource-efficient processing of the input data and thus enable the intra-operative usage of the model, the feature map dimensions at each sub-module were reduced. The resulting input and output resolutions of each sub-module’s feature maps of the 3D YOLOv3 architecture are presented in Table 1. As can be seen, the input volumes’ resolutions were reduced to 160×160×160  voxels. As a consequence, the resolutions of all three scale predictions were reduced to 5×5×5  voxels, 10×10×10  voxels, and 20×20×20  voxels.

**Table 1:** Adapted YOLO architecture for volumetric data.

Block	Input resolution	Output resolution	# In Ch	# Out Ch
CNN1	160×160×160	160×160×160	1	32
CNN2	160×160×160	80×80×80	32	64
RES1	80×80×80	80×80×80	64	64
CNN3	80×80×80	40×40×40	64	128
RES2	40×40×40	40×40×40	128	128
CNN4	40×40×40	20×20×20	128	256
RES3	20×20×20	20×20×20	256	256
CNN5	20×20×20	10×10×10	256	512
RES4	10×10×10	10×10×10	512	512
CNN6	10×10×10	5×5×5	512	1024
RES5	5×5×5	5×5×5	1024	1024
CNN7	5×5×5	5×5×5	1024	512
CNN8	5×5×5	5×5×5	512	1024
SPD1	5×5×5	5×5×5	1024	512
	5×5×5	5×5×5	1024	29
CNN9	5×5×5	5×5×5	512	256
UPS1	5×5×5	10×10×10	256	768
CNN10	10×10×10	10×10×10	768	256
CNN11	10×10×10	10×10×10	256	512
SPD2	10×10×10	10×10×10	512	512
	10×10×10	10×10×10	512	29
CNN12	10×10×10	10×10×10	512	128
UPS2	10×10×10	20×20×20	128	384
CNN13	20×20×20	20×20×20	384	128
CNN14	20×20×20	20×20×20	128	256
SPD3	20×20×20	20×20×20	256	29

The underlying algorithm’s goal is to detect classified axes aligned with bounding boxes. For the 3D case, the labels contain three values (x,y,z) describing the object’s position (center) and three values (w,h,d) describing the bounding box’s width, height, and depth. All of these parameters are normalized with respect to the volume’s dimensions and lie within the range of [0,1].

Furthermore, each ground truth bounding box contains the class value (c) of its associated vertebra: c=1 for cervical, c=2 for thoracic, and c=3 for lumbar.

In addition to these parameters, the orientation for each object (vertebra) in terms of their standard planes is extracted. Our group previously showed that simultaneous regression of the plane spanning vectors gives better results than using Euler angles or quaternion representation.^6^ Therefore, the spanning vectors of the three planes were added to the parameter set. To incorporate the plane spanning vectors in the training, the loss function was extended by the mean squared error between the predicted and ground truth values for each box containing an object. This results in the following loss function:

$$L=w_{\mathrm{OL}}*\mathrm{OL}+w_{\mathrm{NOL}}*\mathrm{NOL}+w_{\mathrm{BL}}*\mathrm{BL}+w_{\mathrm{CL}}*\mathrm{CL}\;+w_{\mathrm{APL}}*\mathrm{APL}+w_{\mathrm{CPL}}*\mathrm{CPL}+w_{\mathrm{SPL}}*\mathrm{SPL},$$ (1)

with the “object” loss (OL), the “no object” loss (NOL), the “box” loss (BL), the “class” loss (CL), the “axial sp” loss (APL), the “coronal sp,” loss (CPL) and the “sagittal sp” loss (SPL). The weighting terms’ respective loss portion $w_{xx}$ are initialized in accordance with Redmon and Farhadi^17^ as $w_{\mathrm{OL}}=w_{\mathrm{CL}}=w_{\mathrm{APL}}=w_{\mathrm{CPL}}=w_{\mathrm{SPL}}=1$ and $w_{\mathrm{NOL}}=w_{\mathrm{BL}}=10$. A lumbar vertebra’s size ranging from 21 mm up to 24 mm accounts for 0.13 up to 0.15 of the input patch size of $160^{3}\;\mathrm{mm}$. Thoracic vertebrae having a size of 17 mm up to 24 mm account for 0.11 up to 0.12, and a cervical vertebra’s size ranging from 8 mm up to 13 mm accounts for 0.05 up to 0.08 of the total size. Consequently the anchor boxes were chosen as [0.15×0.15×0.15] for scale 1, [0.11×0.11×0.11] for scale 2, and [0.07×0.07×0.07] for scale 3. Doing so, the anchor boxes represent the most common sizes for each vertebra class. During inference, YOLO uses the object loss and Jaccard index of the bounding box with the current cell to remove unlikely proposals. In addition to these metrics, we propose adding a threshold for the class probability for further removal of false proposals.

### 2.2 Segmentation-Based Algorithm (U-Net)

The most recent state-of-the-art algorithm for multi-object plane regression is the two-step algorithm proposed by Thomas et al.^2^ In the first step, the object of interest is detected. A segmentation network performs this task on a down-sampled version of the volume as the input. Then, flat cylinders representing the standard planes are segmented in an area around the object using the full resolution of the data. Despite its good position and angle regression results, the run time is quite long as several segmentation tasks are executed. Therefore, we simplified the algorithm and skipped the extra localization step. The segmentation network is trained such that cylinders representing the three standard planes of the vertebral bodies are retrieved. The center of gravity of the cylinder coincides with the center of the spinal canal. For each standard plane of one vertebra, such a flat cylinder is created with the basis having the same normal vector as the respective standard plane. The radius and height of the cylinders differ by a factor of 4(d=4·h). For one volume, the size of the cylinders is constant, and it was chosen so that the cylinders do not overlap. This allows the network to segment the vertebrae as individual objects that do not overlap with those of another vertebra. The nnU-Net architecture was modified such that the axial, coronal, and sagittal planes were retrieved in individual channels. The label type is encoded as the label value. This approach enables overlapping labels for the different planes of one vertebra. The loss term for the training was extended to be the sum of the single losses for the three channels, which were calculated following Issensee et al.^18^ as

$$L=w_{\mathrm{CE}}*\mathrm{CE}+w_{\mathrm{DC}}*\mathrm{DC},$$ (2)

with CE denoting the cross-entropy and DC denoting the Dice loss. $w_{\mathrm{CE}}$ and $w_{\mathrm{DC}}$ were set to 1. For the plane parameter calculation, the segmentation masks are clustered using the density-based spatial clustering of applications with noise (DBSCAN) algorithm.^19^ The DBSCAN algorithm does not require information about the number of existing clusters within the data. This enables it for the use on the spine volumes at hand for which the number of existing vertebrae is unknown during prediction. Furthermore, the algorithm directly executes an outlier handling by distinguishing between clusters and noise. After extracting all real clusters, these are thresholded based on half of the number of voxels within the largest existing cluster. This is done to reduce the number of false-positive predictions to maintain only the correct predicted clusters. Thereafter, intersecting clusters for axial, coronal, and sagittal planes are grouped to one vertebra. Then, the approximated center of a vertebra i is calculated as the mean centroid of the related clusters as

$$c_{\text{vertebra}}^{i}=\frac{cc_{\text{axial}}^{i}+cc_{\text{coronal}}^{i}+cc_{\text{sagittal}}^{i}}{3}.$$ (3)

Finally, the standard plane normals are calculated using the principal component analysis (PCA) algorithm.

### 2.3 Data Augmentation

The following data augmentations are applied on the fly during training to expand the number of samples as well as increase the robustness of both approaches to anatomical or scanner-induced discrepancies in the field.

4. **Rotation and scaling**

Rotation and scaling are applied together to reduce the number of needed interpolations and improve the computation speed. The probability for only doing scaling is set as 0.16, for doing only rotation is 0.16, and for executing both is 0.08. The rotation angles for each axis are drawn from U(−30  deg,30  deg). The scaling factor is sampled from U(0.7,1.4).

2. **Gaussian noise**

With a probability of 0.15, zero centered additive Gaussian noise is added. The variance of the noise is drawn from U(0,0.1).

3. **Gaussian blur**

The blurring augmentation is applied with a probability of 0.2. The width in voxels of the used Gaussian kernel σ is sampled from U(0.5,1.5).

4. **Brightness**

With a probability of 0.15, the intensity values are multiplied by a factor x sampled from U(0.7,1.3).

5. **Contrast**

With a probability of 0.15, the intensity values are multiplied by a factor x sampled from U(0.65,1.5). Afterward, the values are clipped to their original value range.

6. **Simulation of low resolution**

With a probability of 0.25, a sample is down-sampled by a factor of U(1,2) using nearest neighbor interpolation and then sampled back up to their original size with cubic interpolation.

7. **Gamma transform**

With a probability of 0.15, the intensity values of a sample are scaled to a factor between [0,1] of their respective value range. Subsequently, a nonlinear intensity transformation of $i_{\text{new}}=i_{\text{old}}^{\gamma }$ is applied per voxel, with γ being sampled from U(0.7,1.5). At last, the intensity values are scaled back to their original value range.

## 3 Experiments

### 3.1 Datasets

For the study, a proprietary dataset consisting of 658 CBCT volumes is used. The volumes cover the cervical spine region (150 volumes), thoracic spine region (130 volumes), and the lumbar spine region (378 volumes). The volumes were acquired intra-operatively using a mobile C-arm system Cios Spin manufactured by Siemens Healthineers. Depending on the spinal region, a volume contains between 1 and 10 vertebrae. The volumes are of size 512×512×512  voxels with 0.313 mm voxel spacing and were reconstructed offline with the Feldkamp-David-Kress algorithm^20^ with parameters equal to the product standard settings. The annotation of the standard planes was done by a medical engineer after 2 hours of training with a trained physician using a syngo XWorkplace VD20^21^ that was modified to store the plane description. During the annotation, the planes per vertebra were coupled and annotated simultaneously to ensure orthogonality among them. An expert physician and a senior medical engineer validated the annotations on a random basis.

### 3.2 Study Design

For the comparison of the detection-based algorithm with the segmentation-based algorithm, we perform a holdout validation. The training set contains 440 volumes, and the test set includes 218 volumes with 1126 ground truth vertebrae.

The following performance measures were evaluated.

**Vertebra detection:** the models’ vertebra detection abilities are measured based on the accuracy, error rate, recall, and precision.

**Vertebra classification:** during labeling, the individual vertebrae were classified only to their spinal region without further specifications. Thus, all cervical vertebrae C1 to C7 were classified to the class “cervical (C),” all thoracic ones T1 to T12 to the “thoracic (T)” class, and all lumbar ones L1 to L5 to the “lumbar (L)” class. This results in a total of three potential classes that a predicted vertebra is assigned to by the model.

The related confusion matrices are created first to evaluate the performance of each model based on their vertebra classification. Afterward, the same metrics as for the vertebra detection are calculated. The classification was only evaluated for correctly detected vertebrae. Thus, the total number of vertebrae differs between the individual models as well as between their confusion matrices.

**Vertebra localization:** to evaluate the models’ vertebra localization abilities, two different methods are used. The first one evaluates the offset of the predicted center with respect to the ground truth center (center-to-center distance ($d_{\mathrm{cc}}$)) for each correctly predicted vertebra. The second method evaluates the distance of the predicted center to each standard plane, respectively (center-to-plane distance). Therefore, this method is separated into the distance from the predicted center to the axial standard plane ($d_{\mathrm{asp}}$), to the coronal plane ($d_{\mathrm{csp}}$), and to the sagittal plane ($d_{\mathrm{ssp}}$). The latter two were further averaged to get a general impression of the center-to-plane distance ($\bar{d_{\mathrm{sp}}}$).

**Vertebra orientation:** to evaluate the alignment of the predicted standard planes, the angle error between the predicted normal and the ground truth normal vector for each standard plane (${∢}_{\mathrm{asp}}$, ${∢}_{\mathrm{csp}}$, ${∢}_{\mathrm{ssp}}$) is calculated. Once again, only the correctly predicted vertebrae were chosen for evaluation.

### 3.3 Implementation

The models are implemented in PyTorch (v.1.6) and trained on Windows 10 systems with 64 GB RAM and 24 GB NVIDIA Titan RTX GPUs. The weights are initialized by the method of Kaiming et al.^22^ The training followed the proposals of the YOLOv3 and nnU-Net, respectively. For the YOLOv3 based approach, the Adam optimizer with learning rate $10^{-5}$ and weight decay $10^{-4}$ was used, and for the segmentation-based approach, a mini-batch gradient descent optimizer with momentum was used. The total number of epochs was set to 1000, verifying the training convergence of all model variants.

## 4 Results

**Fig. 4:**
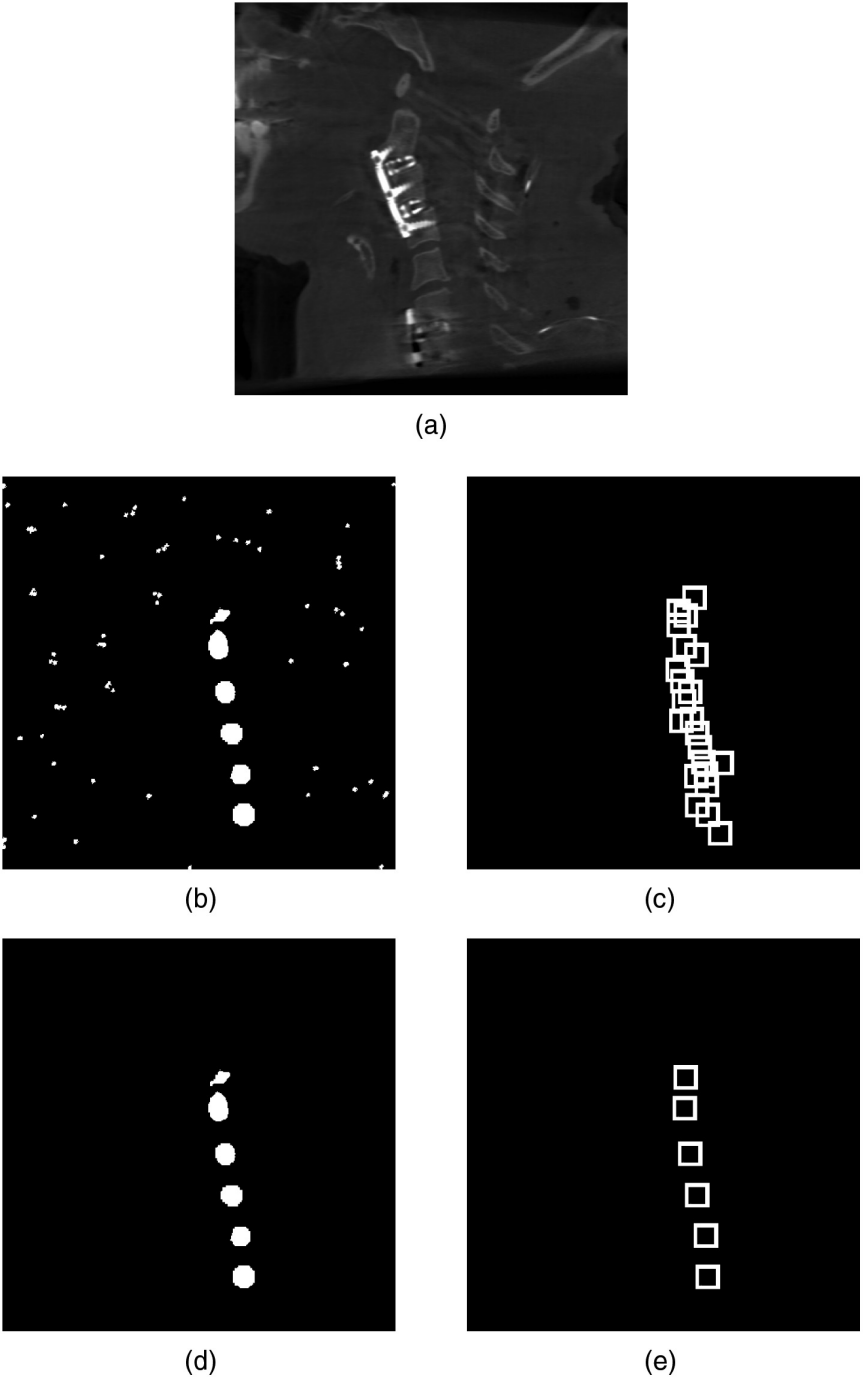
Final prediction results. (a) The orignal volume, The predictions by the U-Net variant (b) before clustering and (d) after clustering. The predictions by the YOLO variant (c) prior to NMS and (e) after NMS.

Figure 4 illustrates the final prediction results for both algorithms. To eliminate false-positive predictions, the segmentation-based approach uses a clustering algorithm for which the clusters are thresholded based on half of the voxel number within the largest existing cluster $\left(\theta =\frac{1}{2}*n_{N}\right)$. All clusters exceeding this threshold are preserved, whereas all smaller ones are omitted. In contrast, the YOLO based approach uses non-max suppression (NMS). The NMS helps to extract the correct vertebra predictions out of the number of total predictions (9125 for the used network configuration of this paper) for each input volume. For that, NMS evaluates each prediction first based on their confidence score. Then, the “intersection over union” (IoU) metric is used to preserve only accurate predictions. As the last step, the predictions’ class probabilities are used to extract the high fidelity predictions. As one can derive from the explanations above, the success of the NMS highly depends on an appropriate choice of the confidence, the IoU, and the class probability thresholds. A different task may require a different configuration of these. Thus, to improve the results for this paper’s task, a hyperparameter optimization was applied to find the optimal threshold combination for all three metrics. The best performance was achieved for the configuration of confidence score=0.10, IoU=0.15, and class probability=0.25.

Table 2 collects the results of the presented YOLO based algorithm and compares them with the ones achieved by the speed optimized segmentation (U-Net)-based approach. Both models are evaluated based on their vertebra detection and classification ability. Then, the localization error and the standard plane parameter prediction accuracy of the correctly determined vertebrae are compared. Finally, the inference times of both approaches are measured.

**Table 2:** Results comparison of both approaches.

		Models			
		YOLO		U-Net	
		μ	σ	μ	σ
**Vertebra detection (%)**	Accuracy	0.91		0.92	
	Error rate	0.09		0.08	
	Recall	0.95		0.93	
	Precision	0.95		0.99	
**Vertebra classification (%)**	Accuracy	0.95		0.96	
	Error rate	0.05		0.04	
	Recall	0.94		0.97	
	Precision	0.94		0.96	
**Vertebra localization** (**mm**)	$d_{\mathrm{cc}}$	2.75	4.08	1.56	1.40
	$d_{\mathrm{asp}}$	0.96	0.77	1.07	1.39
	$d_{\mathrm{csp}}$	1.29	3.75	0.67	0.40
	$d_{\mathrm{ssp}}$	1.74	1.82	0.50	0.26
	$\bar{d_{\mathrm{sp}}}$	1.26	1.85	0.74	1.00
**Vertebra** **orientation (deg)**	${∢}_{\mathrm{asp}}$	5.08	5.70	3.30	1.88
	${∢}_{\mathrm{csp}}$	5.59	5.93	5.36	3.06
	${∢}_{\mathrm{ssp}}$	5.15	7.43	5.50	3.04
	$\bar{{∢}_{\mathrm{sp}}}$	5.00	6.51	4.73	3.67
**Inferencetime** (***s***)	$T_{\min}$ - $T_{\max}$	5		17 to 38	

Table 2 shows that the U-Net-based model performs slightly better than the YOLO based one. The vertebra detection and classification are more accurate. Also, the vertebra localization and orientation are more precise.

However, this difference is small. The YOLO-based model’s localization is worse by 1.19 mm for the center-to-center distance and 0.52 mm for the average center-to-plane distance. Concerning the average angle error between predicted and ground truth plane normals, the YOLO model performs worse by 0.27 deg.

In contrast, comparing both models’ inference times, the YOLO based model surpasses the segmentation-based model. One factor is the absent decoder structure, which enables the YOLO network to process a volume faster. Then, the post-processing of the YOLO based algorithm is much more efficient because no clustering operations or PCA need to be performed. The segmentation-based approach requires these two steps. Therefore, the YOLO-based algorithm achieves its results in about 5 s. This implicates a reduction of computation time by 12 to 33 s compared with the proposed U-Net variant.

## 5 Discussion

The results show that the proposed YOLO-based algorithm yields comparable results to the adapted segmentation-based approach presented by Thomas et al.^2^ Our evaluation confirms the finding of Thomas et al.^2^ that a segmentation-based approach can yield slightly better results concerning the center-to-plane error and the mean angular error. (Note that related work was applied on a different dataset and anatomy. Hence, the results are not directly comparable.) However, this gain is obtained at the cost of computation time. In the application of Thomas et al.,^2^ which implements a comparison of the left and right ankles, the accuracy is crucial. By contrast, in ordinary viewing applications, the user desires a low computation time and tolerates minor plane adjustment errors.

As already pointed out in the introduction, the main benefit of the proposed approaches is that they can predict the standard planes, not for a single point of interest but for an unknown number of vertebrae with relatively small distances to each other within a volume. This influences the prediction process, narrows down the post-processing possibilities, and thus affects the segmentation-based and YOLO-based approaches, as discussed in the following.

As indicated by the high precision values of the approaches’ object detection evaluations, both algorithms result in a high number of true-positive predictions with a simultaneous low number of false-positive predictions (FPs). In detail, for the segmentation-based approach, 1.0% of all predictions are FPs, whereas for the YOLO based approach, FPs accounts for 5.4%.

**Fig. 5:**
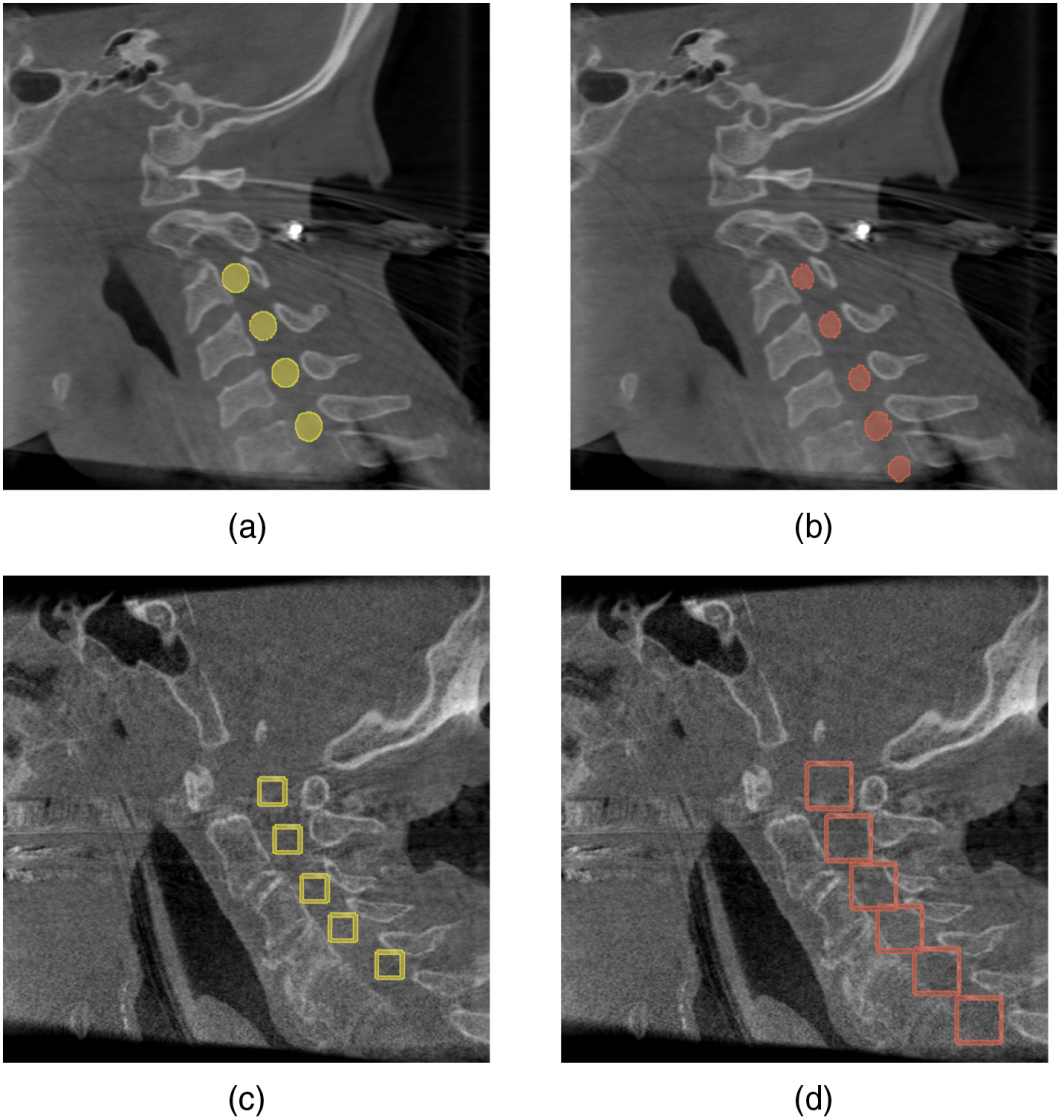
Networks’ predictions for missing ground truth vertebrae. (a) The ground truth segmentation and (b) the predicted segmentation by the U-Net variant. (c) The ground truth bounding boxes and (d) the predicted bounding boxes by the YOLO variant.

However, Fig. 5 shows that these numbers have to be considered carefully, and the true number of FPs is even lower than this. The figure further shows that both approaches predict vertebrae correctly that were not labeled within the ground truth data. This lack of labels results from some vertebrae being omitted from the labels. For these, specific factors such as visual artifacts disabled the generation of precise labels. Also, vertebrae that were only partially contained within a particular volume could not be labeled consistently. Thus, the ground truth labels do not contain an entry for these specific vertebrae.

However, the developed networks of both presented approaches manage to predict those omitted ground truth vertebrae to some extent. Thus, for each prediction of one of those, the number of FPs is increased, in addition to the network predicting a real vertebra.

**Fig. 6:**
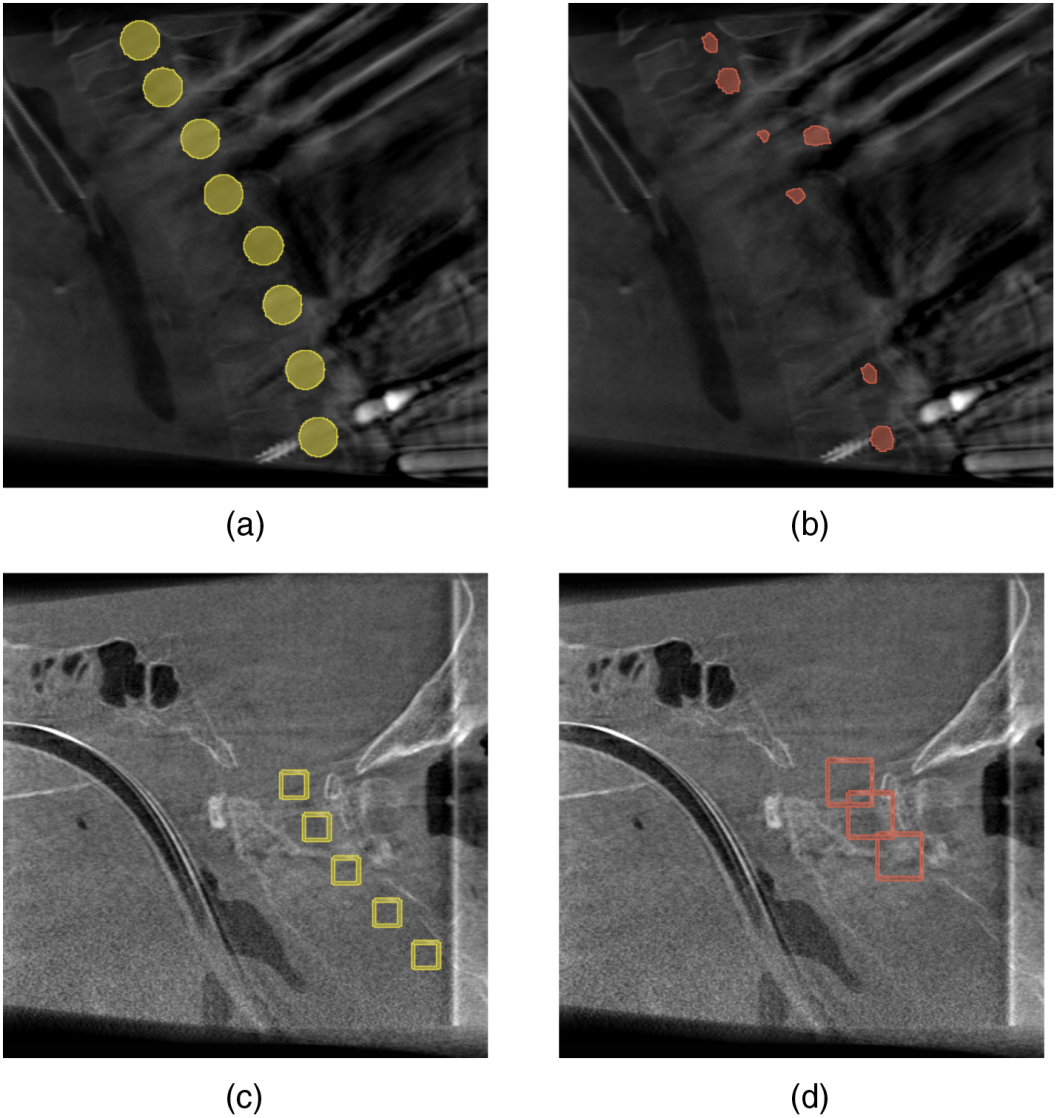
Missed predictions caused by substantial artifacts. (a) The ground truth segmentation and (b) the predicted segmentation by the U-Net variant. (c) The ground truth bounding boxes and (d) the predicted bounding boxes by the YOLO variant.

Although the models are able to compensate for modest artifacts, severe artifacts interfere with the approaches in the creation of appropriate prediction results. It has been observed that artifacts modifying a volume’s intensity values substantially enough cause the networks to fail in their vertebra detection task. Figure 6 presents such an example for each of the two approaches.

It can be seen that both approaches miss the prediction of specific vertebrae. Furthermore, the segmentation-based approach not only misses vertebrae, but it also predicts false positive vertebrae. Thus, such artifacts caused by metal implants such as screws and plates can hinder the presented approaches from functioning correctly.

As can be seen within image (d) of Fig. 5 and Fig. 6, the predicted bounding boxes created by the YOLOs-based approach are larger in size compared with their ground truth counterparts. This size difference between prediction and ground truth applies not only to the presented cervical region but to the entire spine. Thus, in general, the predictions made by the presented network configuration result in oversized bounding boxes. Although these do not affect the predicted standard plane parameters, they influence the extraction of the best predictions out of the total number of individual predictions. In principle, the ground truth bounding box sizes were chosen to avoid intersection and overlapping of bounding boxes of adjacent vertebrae. Thus, accurate predictions of these would likewise result in non-intersecting bounding boxes and would not influence the IoU metric of the applied NMS. However, the prediction of oversized bounding boxes results in the intersection of the individual boxes around every single vertebra. This affects the IoU metric and leads to the potential discarding of precise bounding box predictions by the NMS, thus resulting in a worse accuracy or even the loss of predictions for specific vertebrae. The regions that are mainly affected are the thoracic and cervical spinal regions, but especially the axis and atlas because these are located closest together. In contrast, the oversized bounding box predictions are still able to avoid intersections within the lumbar region due to the larger distances between the associated vertebrae within this region.

One possibility to challenge the discarding of proper predictions due to the overlapping of their associated bounding boxes would be the adjusting of the IoU metric’s threshold to retain more predictions. However, this leads to the retention of more redundant predictions, which increases the number of FPs and aggravates the results at the same time. Therefore, to improve the performance of the presented algorithm, the predicted bounding box sizes have to be reduced. This can be established by increasing the number of anchor boxes per level to more precisely represent the different vertebrae sizes. Currently, there is only one anchor box size for each vertebra. However, vertebrae sizes of one spinal region differ among the individual volumes. Thus, having more anchor box configurations for one particular region to cover the different sizes more efficiently would help to decrease the predicted bounding box sizes. However, having more anchor boxes per scale would also increase a network’s number of total predictions, affecting the inference time. Therefore, it has to be evaluated if the performance improvement is worth the increase in inference time.

Other ideas customizing the ground truth bounding box generation, such as establishing a uniform size for all vertebrae instead of the current volume respective generation, will not improve the results. A uniform size inspired by the cervical vertebrae would result in boxes that are too small for an appropriate representation of the thoracic and lumbar ones. By contrast, a size rested on the dimensions of the lumbar vertebrae would lead to boxes being too large for the thoracic and cervical ones. A size reflecting the thoracic vertebrae instead would neither benefit the representation of the cervical nor the lumbar vertebrae. Likewise, specifying the ground truth bounding box size depending on the region affiliation of the respective vertebra instead of a uniform size would not improve the results either. Although this approach incorporates more information by including the vertebra type for the generation of its bounding boxes in the first place, it lacks adaptability to the high variance of different spine appearances. A taller person has larger vertebrae than a smaller one, and in particular, a child has smaller vertebrae than an adult. Furthermore, spinal injuries or degeneration cause the alteration of vertebrae sizes as well as the modification of the space between neighboring vertebrae. Thus, a predetermined ground truth bounding box size would disable the dynamical adjustment of the bounding boxes to the variation within the used volumes similar to how it has been done so far.

## 6 Conclusion

Mobile C-arm systems are used within the intra-operative environment to allow for 3D assessment during the intervention. However, to enable this, the acquired 3D volumes must first be aligned with the patient’s anatomy. This alignment is based on the standard planes of the respective anatomical region. After extracting the standard plane parameters, the volume is rotated respectively to restore a standardized view of the anatomy. The idea of this work was to develop an approach that extracts the standard planes faster than the current manual procedure using deep learning methods. For that, we have presented, to the best of our knowledge, the first YOLOv3-based network to automatically localize the standard planes of vertebral bodies within intra-operative CBCT volumes. We depicted how we adapted the original parameters to be within today’s hardware constraints for training of such a network. For comparative purposes, the algorithm’s performance was evaluated against a state-of-the-art segmentation-based approach.

In conclusion, the proposed algorithm based on the Yolov3 architecture showed similar good results compared with the segmentation-based algorithm. The latter method has its benefits when accuracy is of great importance. In a surgeon’s daily life, the observed slightly worse results are negligible. However, the speed gain resulting in a run time of 5 s makes the algorithm suitable for use in an intra-operative scenario.
